# Post-COVID-19 chronic inflammatory demyelinating polyneuropathy: a case report of diagnosis and management

**DOI:** 10.1097/MS9.0000000000004827

**Published:** 2026-04-01

**Authors:** Osaide W. Amro, Omar E. Salah, Ali A. Doudin, Hussam Y. Salem, Anas R. Tuqan, Ruba Saadeh

**Affiliations:** Faculty of Medicine, Al-Quds University-School of Medicine, Jerusalem, Palestine

**Keywords:** case report, CIDP, COVID-19, demyelination, peripheral neuropathy

## Abstract

**Introduction and Importance:**

Chronic inflammatory demyelinating polyneuropathy (CIDP) is an autoimmune disorder characterized by demyelination and axonal damage in peripheral nerves, leading to progressive weakness and sensory impairment. Neurological complications have been reported in multiple COVID-19 cases, ranging from mild symptoms such as headaches to severe manifestations like demyelination and stroke.

**Case Presentation:**

We report the case of a 20-year-old female who initially presented with feverish sensation, non-productive cough, generalized fatigue, arthralgia, and muscle pain. She tested positive for SARS-CoV-2 via polymerase chain reaction and received supportive treatment with home isolation for 20 days. While most symptoms resolved, muscle pain persisted. Over time, the patient developed progressive muscle pain and constant weakness in both upper and lower limbs, worsened by repetitive movement. Additional neurological symptoms included symmetrical foot drop, frequent falls, tremors, and difficulty performing fine motor tasks. Laboratory findings and nerve conduction studies were consistent with CIDP based on established diagnostic criteria. The patient received intravenous immunoglobulin therapy and had residual disability.

**Clinical Discussion:**

Several case reports have associated CIDP with various infectious agents. With the advent of the COVID-19 pandemic, there has been an emergence of CIDP cases following SARS-CoV-2 infection or vaccination. This case adds to the growing body of evidence suggesting a link between COVID-19 and autoimmune neurological complications such as CIDP.

**Conclusion:**

This case highlights a rare instance of post-COVID-19 CIDP, underscoring the importance of considering autoimmune neuropathies in the differential diagnosis of patients presenting with progressive neuromuscular symptoms after COVID-19.

## Introduction

Chronic inflammatory demyelinating polyneuropathy (CIDP) is an autoimmune neurological disorder characterized by either a relapsing–remitting or a progressive course, where the immune system mistakenly targets and damages the myelin sheath and axons of peripheral nerves^[^1^]^. The clinical presentation typically includes progressive or recurrent symmetrical weakness, paresthesia, sensory impairments, and diminished or absent tendon reflexes in the extremities, evolving over a period of at least 2 months^[^2^]^. While COVID-19 (SARS-CoV-2) primarily manifests as a respiratory viral infection, it is associated with a range of extrapulmonary and neurological complications. Researches have indicated that COVID-19 patients can develop neurological symptoms, which can vary from mild manifestations such as headaches, myalgias, anosmia, and ageusia, to more severe conditions, including encephalitis, neuropathy, demyelination, and stroke^[^3^]^.

HIGHLIGHTS

- Physicians should monitor for post-COVID neurological complications such as muscle weakness, foot drop, and tremors, which may indicate chronic inflammatory demyelinating polyneuropathy.
- Delayed diagnosis and multiple misdiagnoses can worsen patient outcomes by prolonging disability.
- Intravenous immunoglobulin therapy has shown effectiveness in improving symptoms, though some patients may retain residual disability.

This case report details the first presumed documented instance of post-COVID-19 CIDP in Palestine, contributing to the growing body of literature on this association. Importantly, no multicenter studies have yet examined the incidence of CIDP following COVID-19 infection or vaccination on a global scale.

This case report has been reported in line with the CARE criteria^[^4^]^.

## Case presentation

In March 2021, a 20-year-old female with a history of polycystic ovary syndrome, for which she was not receiving treatment, was admitted to the hospital with complaints of fever, nonproductive cough, generalized fatigue, arthralgia, and muscle pain that had begun 1 day prior to admission. Physical examination revealed no remarkable findings. A SARS-CoV-2 polymerase chain reaction test was performed and returned positive. Following 20 days of home isolation and supportive care, her flu-like symptoms resolved; however, the muscle pain persisted. She reported no significant family medical history and was not taking any regular medications.

Over the subsequent months, the patient experienced a progressive worsening of muscle pain, eventually developing into diffuse, constant muscle weakness in both upper and lower extremities, exacerbated by repetitive movements. Additional symptoms included symmetrical foot drop, frequent falls, tremors, and difficulty performing fine motor tasks. During this time, she sought multiple medical evaluations and underwent several investigations including head and spinal MRI, laboratory tests (CBC, Chemistry, Thyroid hormones), and vitamin levels, all of which were within normal limits (Table 1).

**Table 1:** Laboratory results summary.

Parameter	Result	Reference range
WBC	10.8	4.6–11 × 10^3^/µL
RBC	4.93	4.1–5.5 × 10^6^/µL
HB	13.3	12–16 g/dL
HCT	39.4	37%–48%
MCV	79.9	80–100 fL
MCH	27.0	27–31.2 pg
MCHC	33.8	31%–35%
PLT	360	140–450 × 10^3^/µL
Lymphocytes	31.7	25%–33%
Monocytes	1.9	3%–7%
Neutrophils	66.4	54%–62%
RDW	13.2	0%–15%
PT (INR)	11.6 (0.9)	11-15 sec
PTT	33 sec	25–55 sec
Creatinine	0.54	0.5–0.9 mg/dL
AST	20	0–37 U/L
ALT	22.3	0–33 U/L
Sodium	141	135–145 mmol/L
Potassium	4.4	3.5–5.3 mmol/L
Chloride	108	98–110 mEq/L
Vitamin B12	287	187–883 pg/mL
Vitamin D (25-OH)	14.1	<20 ng/mL (deficient)
HbA1c	4.8	0%–6.4%
TSH	0.784	.35-4.94 mIU/ml

ALT: alanine aminotransferase. AST: aspartate aminotransferase. HbA1c: glycated hemoglobin. HB: hemoglobin. HCT: hematocrit. INR: International Normalized Ratio. MCH: mean corpuscular hemoglobin. MCHC: mean corpuscular hemoglobin concentration. MCV: mean corpuscular volume. PLT: platelets. PT: prothrombin time. PTT: partial thromboplastin time. RBC: red blood cells. RDW: red cell distribution width. TSH: thyroid-stimulating hormone. WBC: white blood cells.

In September 2022, she was re-evaluated for persistence and progression of her symptoms, cerebrospinal fluid (CSF) analysis revealed albuminocytologic dissociation (Table 2). Neurological examination showed absent deep tendon reflexes bilaterally (biceps, triceps, patellar, and ankle) with muscle power graded 5/5 in all four limbs except for bilateral dorsiflexion at the ankles, which was graded 3/5, accompanied by bilateral foot drop. Sensory and cranial nerve exams were normal, and Romberg’s sign was negative.

**Table 2:** CSF analysis results.

Parameter	Result	Reference range
Appearance	Clear	Clear
Total CSF protein	70 mg/dL	15–45 mg/dL
Glucose	55 mg/dL	45–80 mg/dL
WBC count	10 cells/µL	0–5 cells/µL
RBC count	2,500 cells/µL	0 cells/µL
Lymphocytes (%)	-	Typically, lymphocyte-predominant in viral/immune conditions.
Culture	Negative	Negative

CSF, cerebrospinal fluid; RBC, red blood cells; WBC, white blood cells.

In October 2022, a nerve conduction study (NCS) demonstrated severe axonal and demyelinating sensorimotor polyneuropathy, findings consistent with the diagnosis of CIDP based on established criteria^[^5^]^. The patient was started on rescue regimen for CIDP, consisting of intravenous immunoglobulin (IVIG) at a dose of 2 gm/kg over 5 days, followed by 1 mg/kg every 3 weeks for a total of four infusions. She reported clinical improvement, with upper limb symptoms and fine motor skills returning to normal with increased walking speed and better performance when climbing stairs.

## Discussion

CIDP is frequently considered the chronic counterpart to acute inflammatory demyelinating polyradiculoneuropathy (AIDP), the most prevalent form of Guillain-Barré syndrome (GBS). The reported prevalence of CIDP ranges from 0.7 to 10.3 cases per 100 000 individuals, with a male predominance and a male-to-female ratio ranging from 1.5 to 4**^[^6^]^.**

CIDP primarily affects adults, with incidence increasing with age, though no definitive predisposing risk factors have been clearly identified^[^7^]^. Unlike AIDP, which is typically self-limited, CIDP is chronic and often responsive to medical therapy. The diagnosis of CIDP requires the presence of symmetrical weakness in both proximal and distal muscles, with gradual progression over at least 8 weeks^[^8^]^. Approximately one-third of patients experience a relapsing–remitting course, with partial or complete recovery between episodes^[^9^]^. While coronaviruses mainly target the respiratory and cardiovascular systems, they may also affect the nervous system via different mechanisms, mainly by molecular mimicry, ACE-2 binding, and cytokine storm^[^10^]^. Notably, the combination of SARS-CoV-2 infection and CIDP has been reported only seven times in the literature for this specific association (Table 3).

**Table 3:** Studies reporting post-COVID-19 CIDP.

Reference	Age (year), sex	Presentation	Latency period	Diagnosis	Treatment	Treatment outcomes
Suri *et al*^[^11^]^	47, M	Rapidly progressive pure motor-flaccid quadriparesis with bilateral facial weakness with inability to stand without support	7 months –COVID infection 17 days –COVID vaccine	CIDP	IVIG 0.4 gm/kg body weight per day for 5 days	Patient had progressive and near-complete recovery of the neurological deficit with no further relapses
Patel *et al*^[^12^]^	69, F	Severe weakness, absent reflexes and persistent pain in the upper and lower extremities	1 month –COVID infection	CIDP	IVIG then gabapentin	Patient continued to have paresthesia and tenderness in all her limbs post-IVIG, which partially responded to gabapentin
Fotiadou *et al*^[^13^]^	52, F	Progressive walking difficulties accompanied by upper and lower limb acral paresthesia	8 weeks –COVID infection	CIDP	IVIG 0.4 gm/kg body weight per day for 5 days	GBS disability scale 1
Fotiadou *etal* ^[^13^]^	62, M	Progressive dysarthria and walking difficulties accompanied by facial and acral paresthesias	19 days –COVID vaccine	CIDP	7 PLEX sessions followed by pulsed corticosteroid therapy with oral dexamethasone 40 mg/d for 4 consecutive days every 4 weeks	(GBS disability scale 1), even though facial diplegia persisted.
Coviello *et al*^[^14^]^	48, M	Painful distal paresthesia in the lower limbs with an ascending course and involvement of the hands	14-days COVID infection	CIDP	Eight sessions of selective apheresis using an immunoadsorption technique	Almost complete resolution of neurological symptoms
Samakouh *et al*^[^15^]^	67, F	Severe weakness in upper and lower extremities	2-month COVID infection	CIDP	IVIG 25 g daily (about 0.4 g per kg) for 5 days	Partial improvement
Bagella *et al*[16]	49, M	Asymmetric bilateral facial weakness, and paresthesias in the tongue and face	16 days—COVID vaccine	CIDP	IVIG 0.4 g/kg/die for 5 days	Complete resolution of left facial palsy with persistence of slight weakness in the right upper facial area. Gait ataxia improved but did not resolve completely. Paresthesias on the lower limbs were disabling and thus treatment with pregabalin and alpha lipoic acid was started with benefit
Amro *et al*(current case)	20, F	Diffuse muscle weakness in both upper and lower extremities and progressive worsening of muscle pain	Few days – COVID infection	CIDP	IVIG 2 gm/kg body weight per day for 5 days, followed by 1 gm/kg every three weeks for a total of four infusions.	Upper limb symptoms and fine motor skill were back to normal with increased walking speed and better performance when climbing stairs.

CIDP, chronic inflammatory demyelinating polyneuropathy; COVID‑19, Coronavirus Disease 2019; F, female; GBS, Guillain–Barré syndrome; IVIG, intravenous immunoglobulin; M, male; PLEX, plasma exchange.

When diagnosing CIDP, clinicians should also consider other chronic demyelinating neuropathies, such as GBS, multifocal motor neuropathy, IgM-associated demyelinating neuropathies, POEMS syndrome, and medication-induced demyelinating neuropathies, as well as hereditary neuropathies that can mimic CIDP, such as Charcot–Marie–Tooth disease and transthyretin familial amyloid polyneuropathy^[^17,18^]^. A diagnosis of CIDP is typically based on clinical presentation, supported by electrodiagnostic findings indicative of peripheral nerve demyelination or, in rare cases, nerve biopsy. In assessing patients, progressive or relapsing–remitting polyneuropathy affecting both motor and sensory axons, accompanied by areflexia, should raise suspicion of CIDP. This suspicion is strengthened when there is a symmetrical weakness affecting both proximal and distal muscles over a course of 8 weeks or more^[^5^]^. Red flags for alternative diagnoses include respiratory muscle weakness, asymmetric weakness, severe tremor, ataxia, significant pain, autonomic dysfunction, and failure to respond to standard treatments such as corticosteroids or IVIG^[^7^]^. While there is no specific laboratory test for CIDP, standard investigations should aim to exclude other conditions. Electrodiagnostic studies, including NCSs and electromyography, are crucial in confirming the diagnosis. In cases where electrodiagnostic findings are inconclusive, CSF analysis showing albuminocytologic dissociation can further support the diagnosis. Although GBS and CIDP share an immune-mediated demyelinating pathophysiology, several features in this case favor CIDP. The patient exhibited a chronic and progressive course of neurological symptoms over several months, whereas GBS typically presents as an acute monophasic neuropathy reaching its nadir within 4 weeks. The persistent and gradually worsening deficits after the initial COVID-19 infection further support CIDP. Additionally, the treatment response aligns with a chronic course, as multiple cycles of IVIG were required for ongoing improvement, unlike the usual single-course response seen in GBS. Finally, electrodiagnostic and CSF findings demonstrated patterns consistent with chronic demyelination rather than the acute demyelinating or axonal changes characteristic of GBS.

The prompt initiation of effective treatment is critical in the management of CIDP. The primary goal is to halt the immune-mediated attack on the myelin sheath of peripheral nerves to minimize axonal degeneration, thereby improving symptoms, function, and preventing long-term disability^[^19^]^. Treatment strategies are tailored based on disease severity and progression. While some patients with mild forms of CIDP may not require treatment, most experience significant impairment and need intervention. In severe cases, multiple treatment trials may be necessary to achieve optimal outcomes^[^5^]^.

First-line treatments for CIDP include IVIG, plasma exchange, and glucocorticoids, all of which demonstrate comparable efficacy and are recommended by the European Academy of Neurology/Peripheral Nerve Society (EAN/PNS) guidelines^[^5^]^. The choice of therapy depends on factors such as disease severity, patient comorbidities, side effects, availability, and cost^[^20^]^. IVIG is often preferred for CIDP patients as it is more convenient compared to plasma exchange^[^5^]^. Corticosteroid therapy is another option, though the optimal regimen remains unclear. Pulsed high-dose corticosteroids using dexamethasone or intravenous methylprednisolone may serve as alternatives to daily prednisone for both initiation and maintenance^[^5^]^.

For severe or refractory CIDP cases, alternative immunosuppressive therapies such as azathioprine^[^9^]^, rituximab^[^21^]^, methotrexate^[^22^]^, cyclosporine, or cyclophosphamide may be considered^[^5^]^. The selection of these treatments depends on factors including the clinician’s familiarity, treatment side effects, disease severity, and patient characteristics. While most CIDP patients respond to initial therapy, approximately 10%–15% are refractory to all first-line treatments. Around 40% may achieve remission or cure, though some may still have residual deficits that are unresponsive to treatment. Additionally, about 18% of patients experience an unstable disease course marked by progression or relapse^[^23^]^. Patients with CIDP should be regularly reassessed, typically every three months after initiating or adjusting treatment and at least semi-annually during maintenance therapy. More frequent evaluations are necessary for those with rapid deterioration^[^5^]^.

## Conclusion

Numerous case reports of CIDP have found associations with multiple pathogenic organisms (Hepatitis B and C viruses, Bartonella henselae, Mycoplasma pneumoniae, Human immunodeficiency virus, Cytomegalovirus and Epstein–Barr virus). With the advent of the COVID-19 pandemic, coronavirus has emerged as another potential trigger for CIDP, with cases reported both post-vaccination and post-infection. This case report presents a unique instance of post-COVID-19 CIDP in Palestine, highlighting the complexities of diagnosis that led to multiple misdiagnoses and significant treatment delays. The diagnostic challenges faced in this case not only heightened the stress associated with the patient’s condition but also resulted in chronic complications that have profoundly affected her quality of life. As a result, she now lives with permanent partial disability, requiring ongoing therapy, experiencing frequent missed workdays, and confronting escalating financial burdens. This case underscores the critical importance of early recognition and timely initiation of treatment for CIDP, which could have mitigated chronic complications and lessened the socioeconomic impact on the patient.

## Data Availability

The data that support the findings of this study are available from the corresponding author upon reasonable request.
